# Gastrointestinal distribution of chicken gastrin-cholecystokinin family transcript expression and response to short-term nutritive state

**DOI:** 10.1016/j.ygcen.2017.10.009

**Published:** 2018-01-01

**Authors:** Angus M.A. Reid, Ian C. Dunn

**Affiliations:** The Roslin Institute, University of Edinburgh, Easter Bush, Midlothian, Scotland EH25 9RG, UK

**Keywords:** Satiety, Avian, Hormone, Feeding, Poultry

## Abstract

The related peptide hormones cholecystokinin (CCK) and gastrin are conserved throughout vertebrate clades and implicated in energy homeostasis. CCK is generally accepted as a satiety hormone in poultry, but the role of gastrin remains poorly studied. Functional dissection of these ligands is required to characterise the molecular control of growth & satiety in the domestic chicken, for which there is an increasingly pressing mandate. There are limited descriptions of physiological distributions for the two genes in birds, and these are mostly reliant on immunohistochemistry which can prove problematic due to the shared structure of the targets. Therefore, we have defined the tissue distributions of CCK and gastrin in the chicken, focussing on the gastrointestinal tract, by using transcript-dependent techniques to improve reliability by increasing specificity. Though considerably more highly expressed in the brain, gastrointestinal CCK transcripts were dispersed throughout the small intestine and particularly around the proximal ileum. Gastrin expression was strictly limited to the gastric antrum region of the intestinal tract, albeit very highly expressed. We demonstrate that CCK mRNA expression does not respond as expected for a short-term satiety hormone, and that the short-term response of gastrin expression is paradoxical compared to its role in mammals. These results partially corroborate previous peptide distribution studies and initiate exploration of the nutrient-responsive roles of these hormones in avian energy balance.

## Highlights

• Anatomical distributions of chicken CCK and GAST mRNA expression are resolved.
• Expressional responses of CCK and GAST to short-term nutritive state are investigated.
• CCK mRNA expression does not respond to acute satiety state.
• GAST mRNA expression is upregulated under short-term fasting.

## 1 Introduction

Recent years have seen increasing interest in the characterisation of avian energy homeostasis, both in order to optimise poultry production and welfare and to better understand endocrine regulation of vertebrate energy balance and evolution of the mechanisms which underlie it. The ‘broiler-breeder paradox’ – restriction of feed intake to maintain reproductive health in broiler parent flocks – is a prominent example of welfare concern arising from intense selective breeding in chickens for meat production. This might be solved or ameliorated if hormonal response to nutrition was better understood and breeding or husbandry managed to prevent aberrant ovarian follicular development (Decuypere et al., 2006). Further concerns surround force-feeding in the production of foie gras, and the need for development of alternatives are currently under debate (Guemene and Guy, 2004, Rochlitz and Broom, 2017). Some steps have been taken to describe how endocrine and neuroendocrine signalling is affected under such atypical feeding conditions in poultry (Boswell et al., 1999, Davail et al., 2003, de Jong et al., 2003, Dunn et al., 2012, Dunn et al., 2013), however much work is yet required to fully understand the molecular control of avian growth and its significance to modern agricultural practice, particularly considering the contrasting characteristics of energy balance mechanisms in birds compared to other vertebrates (Honda et al., 2017).

The gastrin-cholecystokinin peptide family comprises the variably processed and modified products of two genes; *GAST* (producing preprogastrin) and *CCK* (producing preprocholecystokinin). Gastrin and cholecystokinin represent one set of hormones relatively well-described in mammals but neglected in birds. Both genes are conserved across vertebrate species, likely arising from a duplication event early in the vertebrate lineage (Johnsen, 1998), and descend from an ancient peptide class conserved throughout metazoans (Dupré and Tostivint, 2014, Yu and Smagghe, 2014). Gastrin and CCK have related physiological roles in vertebrates, being heavily implicated in peripheral signalling to regulate appetite and digestive organ activity, as well as in emotion and behaviour (Ballaz, 2017). Products of both genes are variably processed to an impressive spectrum of molecules, relative abundances of which are dependent on species, tissue dietary composition, and specific degradation rates among other factors, as comprehensively summarised by Guilloteau et al. (2006). All CCK and gastrin molecules have similar C-terminal structures and bind a common receptor (CCKBR) with similar efficacy dependent on sulphation at the C-terminus-proximal tyrosyl residue whereas CCKAR is only practically bound by tyrosyl-sulphated CCK (Huang et al., 1989, Guilloteau et al., 2006). This posttranslational complexity undermines the validity of immunological studies employing antibodies raised against certain molecular forms. Common physiological effects seem to be conferred by all functional products of each gene (Guilloteau et al., 2006), so studies on the gene transcript may be more reliable and will complement the interpretation of existing studies which used immunological tools.

The basic gastrointestinal distributions of CCK and gastrin transcript and peptides have been described in chickens (Martinez et al., 1993, Honda et al., 2017), however these studies either lack resolution or are dependent on antibodies as discussed. Likewise, although some work has been carried out to assess the function of CCK as a regulator of appetite (Tachibana et al., 2012), stimulation of acid secretion by gastrin (Campbell et al., 1991, Furuse and Dockray, 1995) and CCK and gastrin as modulators of gastrointestinal motility (Martinez et al., 1993b3), the response of native gastrin and CCK expression to disparate nutritive states in birds has not been addressed. We therefore set out to better describe the anatomical distribution of CCK and gastrin production, and how their expression is affected by short-term hunger and satiety states in the domestic chicken.

## 2 Materials and methods

### 2.1 Animal Material

Use of animals was approved by the Roslin Institute Animal Welfare and Ethical Review Body and experiments were carried out under the Animals (Scientific Procedures) Act 1986, project licence 70/7909.

#### 2.1.1 Distribution of gastrin and CCK expression

In order to assess the distribution of expression of gastrin and CCK in chicken tissues by qPCR, four Lohmann Classic hens reared in standard conditions were killed by barbiturate overdose at peak of lay and a range of tissue samples was collected from intestine, visceral organs, brain and musculo-skeletal tissue. Material for *in situ* hybridisation was harvested from broiler breeders reared in standard conditions with commercial food restriction to achieve the breeding company’s target growth rate (Aviagen, 2013) until 11 weeks of age when birds were moved to individual cages. Following a 5-day cage acclimatisation period, birds were fed either *ad libitum* or continued commercial restriction for a further 2.5d before cull by barbiturate overdose. The antrum was dissected to include part of the gizzard and duodenum at either side. A section of proximal ileum just posterior to the vitelline diverticulum was also dissected. All samples were snap-frozen on dry ice.

#### 2.1.2 Response to short-term nutritive state

To characterise the responses of gastrin and CCK to short-term hunger and satiety, 50 NOVOgen brown birds were sexed by genotyping (Clinton et al., 2001) at 2d and reared to 6d in a single floor pen before being split into four floor pens; two containing males (n = 14/pen), and two containing females (n = 11/pen), balanced by bodyweight for each sex. *Ad libitum* feeding was provided until 16d, temperature was 26 °C, light was 14L:10D with lights-on at 07:00, and all birds were handled daily for 4 days prior to cull at 17d. Feed was removed from all pens at 05:00 on the day of cull, and reintroduced to one pen of each sex after 3 h (08:00). The remaining pens maintained fast for the remainder of the experiment. 2.5 ± 0.5 h of feed after reintroduction of feed or maintenance of fast (10:00–11:00), five females and seven males from each treatment were culled. All remaining birds were culled 7.5 ± 0.5 h after reintroduction of feed or maintenance of fast (15:00–16:00). Chicks of this age have an average whole digestive tract transit time of 2.65 ± 0.05 h (mean ± SEM, ingestion to defecation, unpublished data), so all gastrointestinal regions of interest should have been in recent contact with nutrients by 2.5 h. All birds were killed by cervical dislocation and immediately dissected to harvest 40–100 mg samples of gastric antrum and proximal ileum, which were snap-frozen on dry ice. All samples were taken in a coronal plane to include all intestinal tissue strata.

### 2.2 Design of oligonucleotide primers and probes

**Fig. 1:**
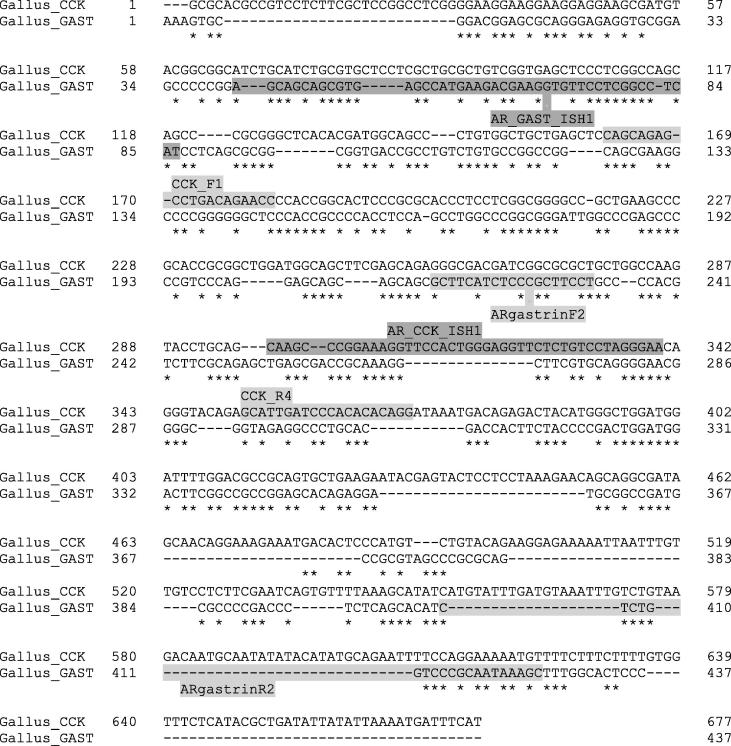
Alignment of CCK and gastrin mRNA sequences. Primer (light grey) and probe (dark grey) annealing positions are indicated to show targeted areas of low shared identity. Further details of primers and probes used in this study can be found in Table 1.

Details of all primers and probes used in this study are summarised in Table 1. Novel primers to amplify chicken preprogastrin (GI:45382320) and chicken CCK (GI:48976040) mature mRNA sequences were designed using Primer3 (Rozen and Skaletsky, 2000, Untergasser et al., 2012). Oligonucleotide probes for *in situ* hybridisation were designed manually to conform to the following parameters: ∼55% GC content (48–62%), ∼45mer length (43–47mer) and melting temperature (T_m_) as high as possible within those parameters and at least 20 °C greater than the highest predicted tertiary structure T_m_ predicted by OligoAnalyzer 3.1 online software (Integrated DNA Technologies). Chicken CCK and gastrin preprohormone mRNA sequences were aligned using MUSCLE (Edgar, 2004) to identify regions that were divergent and to avoid selecting regions of similarity between the two transcripts for targeting oligonucleotide primer and probe annealing (Fig. 1). Similarity was calculated for each probe against the unintended target mRNA reverse-complement by the Smith-Waterman algorithm using EMBOSS Water (Smith and Waterman, 1981, Rice et al., 2000) and found to be 48.9% for AR_GAST_ISH1 and 60.9% for AR_GAST_ISH1. BLASTN (NCBI) returned no unintended chicken targets for either probe. Primers for quantification of LBR, YWHAZ and NDUFA1 as reference genes were described previously (Reid et al., 2017). Sigma-Aldrich UK supplied all oligonucleotide primers and probes.

**Table 1:** Details of oligonucleotide primers and probes.

Oligonucleotide name	Type	Sequence (5′-3′)	qPCR target & amplicon length
CCK_F1	Primer	CAGCAGAGCCTGACAGAACC	NM_001001741.1 210bp
CCK_R4	Primer	CCTGTGTGTGGGATCAATGC	
ARgastrinF2	Primer	GCTTCATCTCCCGCTTCCT	NM_205400.1 212bp
ARgastrinR2	Primer	GCTTTATTGCGGGACCAGAG	
YWHAZ_F	Primer	GTGGAGCAATCACAACAGGC	NM_001031343.1 223bp
YWHAZ_R	Primer	GCGTGCGTCTTTGTATGACTC	
LBR-F	Primer	GGTGTGGGTTCCATTTGTCTACA	NM_205342.1 80bp
LBR-R	Primer	CTGCAACCGGCCAAGAAA	
NDUFA1-F1	Primer	ATGTGGTACGAGATCCTGCC	NM_001302115.1 203bp
NDUFA1-R1	Primer	TTCTCCAGACCCTTGGACAC	
AR_CCK_ISH1	Probe	TTCCCTAGGACAGAGAACCTCCCAGTGGAACCTTTCCGGGCTTG	–
AR_GAST_ISH1	Probe	ATGAGGCCGAGGAACACCTTCGTCTTCATGGCTCACGCTGCTGCT	–

### 2.3 Preparation of cDNA

Total RNA was isolated from tissue homogenised in TRIzol reagent (Invitrogen) using the Direct-zol RNA Kit (Zymo Research) to manufacturer’s specifications, with in-column DNase treatment. 1μg total RNA per sample was reverse transcribed using the High Capacity Reverse Transcription Kit (Applied Biosystems) in 20 μl reactions according to manufacturer’s guidelines and the product diluted to 110 μl total volume per sample with water.

### 2.4 Quantitative polymerase chain reaction (qPCR)

Brilliant III Ultra-fast SYBR Green qPCR Mastermix and the Mx3005p qPCR System with MxPro software (Agilent Technologies) were employed according to the manufacturers’ guidelines and as described previously (Whenham et al., 2015). Briefly, 10 μl SYBR mix, 8 μl cDNA product, 0.4 μl 20 μM forward primer, 0.4 μl 20 μM reverse primer, 0.3 μl 1/500 rox reference dye solution and 0.9 μl H_2_O were mixed for each 20 μl reaction. Thermal conditions were consistent for all assays: 50 °C; 120 s, 95 °C; 120 s, (40 cycles of 95 °C; 15 s, 60 °C; 30 s), then 95 °C; 60 s, 60 °C; 30 s, 95 °C; 15 s. Apparent reaction efficiencies were between 96and 99%, as determined by analysis of the standard dilution curve. Amplicons were bidirectionally sequenced using LightRUN Sanger sequencing (GATC Biotech) to confirm identity. LBR, NDUFA1 and YWHAZ were chosen as reference genes due to their reliability in previous avian studies (McDerment et al., 2012, Olias et al., 2014) and quantified as above. Normalisation was achieved by dividing the raw expression value for the gene of interest by the geometric mean of the LBR and YWHAZ raw expression values.

### 2.5 *In situ* hybridisation

*In situ* hybridisation employed reagents and protocol as described previously (Meddle et al., 2007). Briefly, oligonucleotide probes specific to mRNAs of interest (see Table 1) were radiolabelled with ^35^S-dATP and incubated overnight with fixed 15 μm tissue sections on polysine slides. Slides were exposed for 14 days in autoradiographic emulsion before development, fixation and haemotoxylin/eosin counterstaining.

## 3 Results

### 3.1 Distribution of gastrin and CCK

**Fig. 2:**
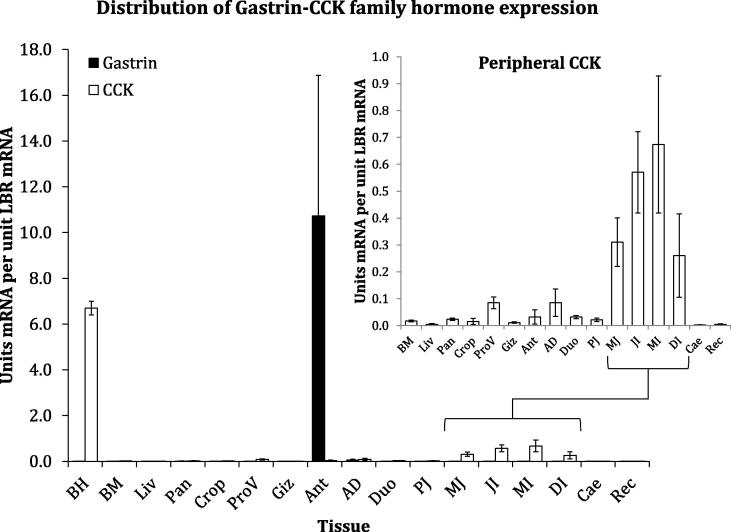
Tissue distribution of chicken Gastrin-CCK hormone family expression. Normalised relative mean (±SEM) gastrin (filled bars) and CCK (open bars) mRNA expression for 17 tissue types in Lohmann Classic brown laying hens (n = 4): basal hypothalamus (BH), breast muscle (BM), liver (Liv), pancreas (Pan), crop, proventriculus (ProV), gizzard (Giz), antrum (Ant), antro-duodenal boundary (AD), duodenum (Duo), proximal jejunum (PJ), mid-jejunum (MJ), jejuno-ileal boundary just distal to the vitelline diverticulum (JI), mid-ileum (MI), distal ileum (DI), caecum (Cae) and rectum (Rec).

Fig. 2 shows distribution of gastrin and CCK mRNA expression levels as assessed by qPCR across a panel of chicken tissues. CCK was found to be primarily expressed in the basal hypothalamus (Fig. 2a), whereas gastrin was exclusively expressed in the gastric antrum region (Fig. 2b). Peripheral CCK exhibited peak expression in the small intestine, particularly around the proximal half of the ileum, with low but detectable expression in other visceral regions, particularly the proventriculus and antro-duodenal boundary regions of the gastrointestinal tract.

**Fig. 3:**
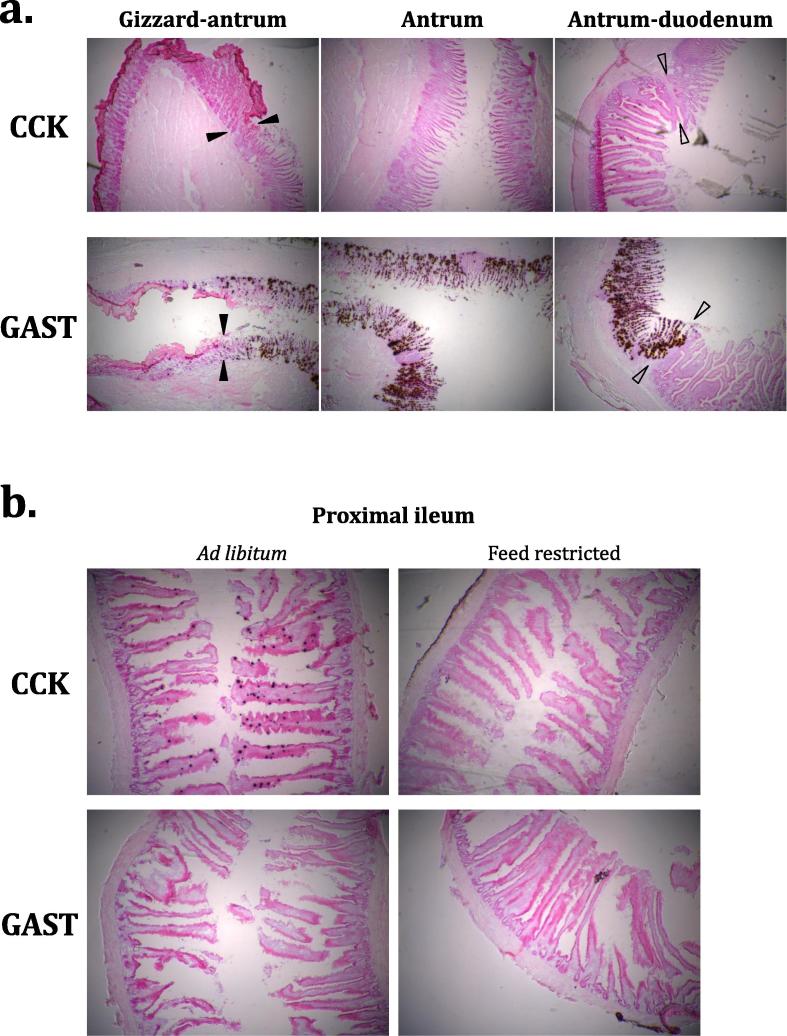
*In situ* hybridisation around the gastric antrum and proximal ileum. 15 μm tissue sections are shown for the gastric antrum in *ad lib*-fed birds (a). Hybridisation signal for CCK (top row) or GAST (bottom row) transcripts. Arrows signify transition from gizzard to antrum (filled) and antrum to duodenum (open). Further 15 μm sections are shown for the proximal ileum in *ad lib*-fed and feed restricted birds (b). Hybridisation signal for CCK (top row) or GAST (bottom row).

Peripheral observations were corroborated by *in situ* hybridisation results which clearly showed a distinct region of high gastrin expression in the antral epithelium (Fig. 3a) but no detectable gastrin in the ileum (Fig. 3b). Discrete high CCK expression was detected in luminal villus cells of the proximal ileum (Fig. 3b) and lower but detectable CCK expression at the proximal duodenum, but not the antrum (Fig. 3a). Notably, both assays agree that antral gastrin mRNA concentration is far greater than ileal CCK mRNA concentration (Fig. 2, Fig. 3). The intensity of ileal CCK hybridisation signal was observed to differ considerably between *ad libitum*-fed and restricted birds (Fig. 3b), but no quantitative analyses were performed for this assay.

### 3.2 Response to short-term nutritive state

**Fig. 4:**
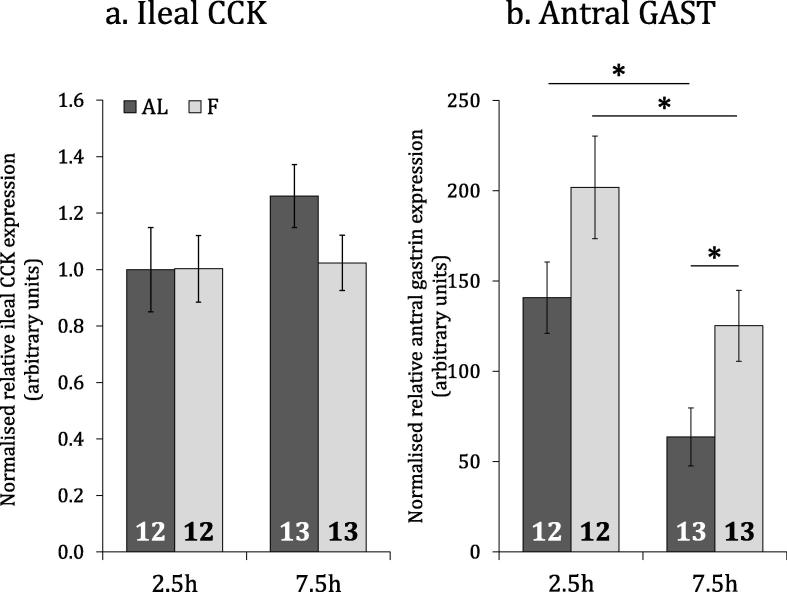
Response of ileal CCK and antral gastrin expression to short-term satiety state. Normalised relative mean (±SEM) ileal CCK (a) and antral gastrin (b) mRNA expression for birds fed *ad libitum* or fasted for 2.5 h and 7.5 h. Number of birds in each group are shown within each bar. Asterisks (*) represent statistical significance at p < .05.

Sex was not found to be a significant factor in any analysis, so data from both sexes are presented together. No significant difference in CCK expression was detected between treatments (F_1,42_ = 0.99, P = .324) or sampling times (F_1,42_ = 1.32, P = .257), and there was no treatment by sampling time interaction (F_1,42_ = 0.96, P = .332) (Fig. 4a). Gastrin expression was higher in the fasted groups compared to the *ad libitum*-fed groups across both sampling times (F_1,42_ = 8.6, P = .005), and lower at the later sampling time compared to the earlier sampling time across both treatments (F_1,42_ = 13.52, P < .001), but there was no interaction between treatment and sampling time (F_1,42_ = 0.00, P = .990) (Fig. 4b).

Notably, across both the distribution assay and the feeding experiment, gastrin expression was found to be far higher than CCK expression in their sites of highest expression (antrum and proximal ileum, respectively) in real terms (i.e., moles of transcript per mg tissue).

## 4 Discussion

Using qPCR and *in situ* hybridisation, we have corroborated and further resolved the results of previous studies of distribution of native gastrin-cholecystokinin peptide family expression in the domestic chicken (Martinez et al., 1993, Honda et al., 2017). Whereas Martinez et al. (1993) employed an immunohistochemical approach (and therefore antibodies which might have been cross-reactive or insensitive to some processed peptide forms), our methods targeted the common mRNA transcript for each gene which allowed greater control of specificity as target regions of low shared identity could be prioritised (Fig. 1). This allowed information on the aggregate expression of the numerous variably processed peptide products of each gene to be inferred, since neither *GAST* nor *CCK* are thought to routinely produce splice variants (Håkanson and Rehfeld, 2002).

CCK was far more highly expressed in the brain than any peripheral region sampled (Fig. 2), which reinforces the role of CCK as an important neuropeptide in birds and is consistent with broad distribution of active CCK peptides (Rehfeld, 2017). This skewed distribution is particularly noteworthy in the context of the recent report that mammalian brain CCK exists almost exclusively in the sulphated form, potentiating activity at the A-type receptor (Agersnap et al., 2016). Of course heightened central expression of CCK does not negate its importance in peripheral regulation of gastrointestinal function, especially since vagal transduction of peripheral CCK influences central energy balance. CCK in the periphery was most highly expressed in the proximal ileum, consistent with murine CCK (Fakhry et al., 2017), but its absolute expression is remarkably low compared to that of gastrin in the gastric antrum. This is interesting as it suggests that the magnitude of paracrine gastrin binding at B-type receptors local to the antrum must be profound in comparison to CCK binding, assuming expression of the transcript translates to peptide release. Chicken gastrin expression is strictly limited to the gastric antrum (Fig. 2), suggesting a specific role in responding to the luminal environment at the transition from gizzard to small intestine. This is in keeping with the gastric acid secretion-regulating function of vertebrate gastrin, as originally demonstrated in the chicken (Campbell et al., 1991). This difference in expression has to be taken in context however, since the total gastrin-expressing intestinal region (the gastric antrum) is very short compared to the tissue expressing CCK, which is effectively most of the small intestine (Fig. 2). Gastrin and CCK seem to have functionally opposite effects on regulation of gastric acid (Guilloteau et al., 2006), however the inhibitory effect of CCK is dependent on signalling via CCKAR (Chen et al., 2004), whereas gastrin acts only at CCKBR, so disparate threshold ligand concentrations for each of these signalling routes might explain this apparent paradox.

Although birds are considered ‘monogastric,’ their gastric lumen is compartmentalised into the proventriculus (glandular stomach) and ventriculus or gizzard (muscular stomach). The proventriculus best resembles the mammalian monogastric stomach in form and function, and so is sometimes referred to as the ‘true stomach’ (Mussehl et al., 1933, Zaher et al., 2012). The strict delineation of avian gastrin within the ‘antrum’ region observed here resembles primary mammalian gastrin production at the pyloric antrum which suggests homology of these gastrointestinal structures between birds and mammals. This provides evidence that the mammalian monogastric stomach can be considered homologous to the entire gastric region in birds (i.e. the gizzard is a specialised compartment of the whole ‘true stomach’ and not for example an adaptated region of intestinal tissue), in approximate keeping with extant belief (Smith et al., 2000, Nielsen et al., 2001). Its strength and fidelity of expression make gastrin a candidate marker for evolutionary comparisons of vertebrate digestive tract physiology.

CCK is heavily implicated in the short-term satiety response in vertebrate species (Havel, 2001, Murashita et al., 2007, Moran, 2009, Gibbons et al., 2016, Honda et al., 2017, Volkoff et al., 2017). The circulating peptide longevity is known to be very short (Liddle et al., 1985), although a delay in transcriptional response might have been expected, as observed for the satiety factor peptide YY in chickens (Reid et al., 2017). CCK did not alter significantly in response to short-term satiety state within the scope of the fed/fasted experiment (Section 2.1.2.), neither at 2.5 h nor 7.5 h post-feeding (Fig. 4a). A caveat remains in that differences in the rate of mRNA translation remain unknown and activity may depend on differential post-translational processing, rather than differential expression (Sayegh et al., 2014). Nevertheless, the observed results imply that CCK expression is not significantly affected by immediate nutrient availability in the chicken. On the other hand, the difference in CCK hybridisation signal between *ad libitum*-fed and feed-restricted birds (Fig. 3b) suggests that anticipatory expression might differ between groups under longer-term nutritional challenge. In addition, very short-term expressional response to feeding, as observed in some fish (Murashita et al., 2007, Volkoff et al., 2017), might have been missed by virtue of sampling times in this design.

Gastrin expression differed significantly between treatments, with fasted individuals exhibiting greater expression compared to their fed counterparts at both sampling timepoints (Fig. 4b). This suggests that the short-term nutrient-responsive regulation of gastrin expression in chickens manifests within 2.5 h and is maintained for at least 7.5 h. The observed trend seems paradoxical; why is gastrin, an accepted vertebrate satiety factor, upregulated under fasting conditions in the chicken? Longer-term conditioning to food availability and heightened expression in anticipation of meal consumption might explain this phenomenon, since these birds were fed *ad libitum* for the entire rearing period before induction to experimental treatment. If this is the case, it might be sensible to consider heightened gastrin expression as a means to maintain peptide stocks for secretion upon anticipated detection of nutrients at the gastric antrum. The idea that gastrin expression might be regulated by conditioning is mimicked in the observation that a strong diurnal pattern is apparently maintained regardless of treatment, with gastrin expression decreasing across the experimental timescale for both treatments (Fig. 4b). Attenuation of gastrin expression throughout the waking day makes inherent sense for the diurnal chicken, since it would be ineffective for an animal to produce much gastric acid during, or shortly before, inactive hours. Considering the regulatory interplay between gastrin and gastric acid production (Campbell et al., 1991), relatively lowered postprandial expression of gastrin might simply be due to the inhibitory effect of gastrin-stimulated gastric acid on production of gastrin itself.

In conclusion, we have demonstrated tissue distribution of gastrin and cholecystokinin gene expression in chicken. CCK expression does not seem to respond to short-term satiety, contrary to some antecedent vertebrate studies. Gastrin expression did alter between fed and fasted treatments, however its expression was paradoxically lower in acute satiety and higher in acute hunger, which might be an artefact of conditioning to *ad libitum* feeding conditions. Further studies of the expressional response of these hormones to nutritive state should consider disparate nutrient availability for longer time periods and periprandial sampling, and might clarify similarities and differences between birds and other vertebrate clades.
